# Age and Alzheimer’s Disease

**DOI:** 10.3390/nu8060372

**Published:** 2016-06-16

**Authors:** Bernard Meunier

**Affiliations:** 1 Laboratoire de Chimie de Coordination du CNRS, 205 Route de Narbonne, 31077 Toulouse Cedex, France; bmeunier@lcc-toulouse.fr; Tel.: +33-5-61-33-31-00; 2 School of Chemical Engineering, Guangdong University of Technology, Higher Education Mega Center, No. 100 Waihuan Xi Road, 510006 Guangzhou, China

A recent review article published in *Nutrients* by Brewer [1] attracted the attention of the readers on the role of copper toxicity as major factor in Alzheimer’s disease. The deregulation of copper homeostasis in the AD brain has been studied over the last decade by several groups, and copper chelators have been proposed as drug-candidates to remove the excess of copper ions trapped by amyloid peptides (see References [2,3,4] for recent reviews). Many factors related to aging have been evoked to explain the deregulation of copper trafficking in AD brain. However, Brewer has proposed that inorganic copper of supplement pills and in drinking water are more damaging to cognition than organic copper in food. In addition, to discard the hypothesis of AD as being an aging disease, a commonly accepted idea, the author mentioned that half of the population of France lived to 60 or older in 1911 with a low prevalence of sporadic AD [1]. This data is the median age at death and does not give a good picture of the number of very old people. Since sporadic AD is developing at the age of 70–85, life expectancy is a more suitable parameter to understand the role of aging in the increasing number of AD patients in many countries. Given that only 12.7% of the population was above 60 years of age in France in 1910 [5], one can understand why sporadic AD was not so developed. Life expectancy for men was 63 in 1950 and 78 years in 2013 in France (see Figure 1 below the schema extracted from Reference [6]), meaning that many people are now reaching the dangerous aging zone for Alzheimer’s disease (see added red rectangle on the scheme).

From the evolution of life expectancy in France since 1740, it is easy to understand why Alzheimer’s disease is now a frontline health problem and was not occurring in the “good old days”.

## Figures and Tables

**Figure 1:**
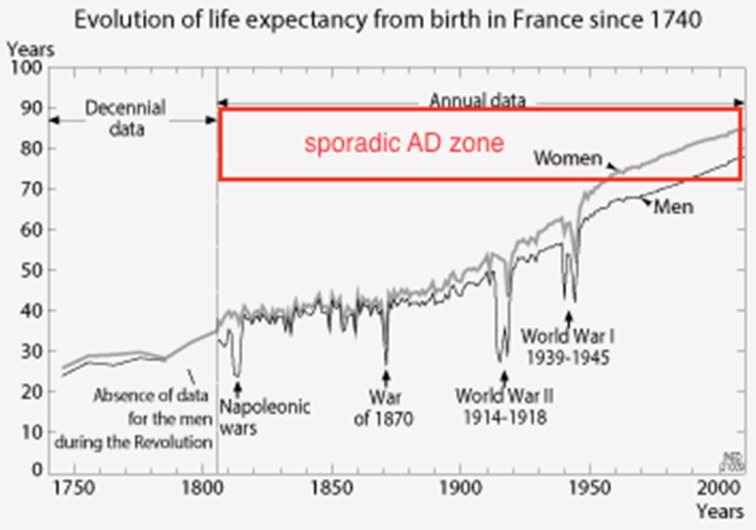
The risk of sporadic AD increases when life expectancy increases.
